# The Multi-Agentization of a Dual-Arm Nursing Robot Based on Large Language Models

**DOI:** 10.3390/bioengineering12050448

**Published:** 2025-04-24

**Authors:** Chuanhong Fang, Xiaotian Yue, Zhendong Zhao, Shijie Guo

**Affiliations:** 1 Academy for Engineering & Technology, Fudan University, Shanghai 200433, China; 22210860103@m.fudan.edu.cn; 2 College of Computer and Control Engineering, Qiqihar University, Qiqihar 161000, China; xtyue@qqhru.edu.cn; 3 School of Artificial Intelligence & Data Science, Hebei University of Technology, Tianjin 300130, China; 202112801012@stu.hebut.edu.cn

**Keywords:** nursing-care robot, large language model, human–robot interaction, multi-agentization, attractor model

## Abstract

Nursing robots are designed to serve users, and their ability to interact with humans, as well as to make task-related decisions and decompositions based on such interactions, is a fundamental prerequisite for autonomous execution of nursing tasks. Large language models offer an effective approach to facilitating human–robot interaction. However, their global perspective can lead to confusion or reduced precision when coordinating the execution of tasks by a dual-arm robot, often generating execution sequences that are inconsistent with real-world conditions. To address this challenge, this study proposes a multi-agent framework, wherein each arm of the nursing robot is conceptualized as an independent agent. Through the application of geometric constraints, these agents maintain appropriate relative positional relationships and achieve coordinated collaboration via a large language model. This approach enhances the task planning capabilities of the robot and improves its efficiency in delivering nursing services.

## 1. Introduction

China is rapidly entering a stage of deep population aging. By the end of 2023, the population aged 60 and above had reached 297 million, with more than 43 million elderly individuals experiencing disabilities, a number that continues to rise [1]. The shortage of caregivers for disabled elderly individuals has become increasingly evident, and there is growing societal anticipation for the practical application of nursing robots [2,3,4]. Humanoid dual-arm nursing robots, characterized by flexible movements and the potential for multifunctional development, have attracted widespread attention [5,6,7]. Since nursing robots are designed to assist disabled or semi-disabled elderly individuals, they must possess a high level of human–robot interaction capabilities [8,9,10].

Large language models (LLMs) can endow robots with more natural and efficient human–robot interaction, as well as enhanced task reasoning and decision-making capabilities [11,12,13]. Ahn et al. from Google [14] leveraged LLMs to analyze the context and intent of language instructions, breaking down complex task commands into executable action sequences for robots. Wu et al. from Princeton University [15,16] focused on household cleaning needs and developed the Tidybot robot, which utilizes language planning and the summarization capabilities of LLMs to infer user preferences widely applicable to human–robot interaction, guiding the robot to pick up and organize cluttered objects in living spaces. The VoxPoser system interprets free-form language instructions and integrates vision–language models to generate 3D value maps, enabling robots to interact with their environment [17].

Establishing prompt strategies that closely align with specific scenarios is crucial for enabling LLMs to understand and generate task-relevant text [18,19]. The University of Southern California and NVIDIA jointly introduced a new model, ProgPrompt [20], which provides LLMs with a Python-based program header as a prompt. This header incorporates action spaces, expected parameters, and environmental information, ultimately generating robot task-planning text that conforms to programming language structures. The Google team developed CaP [21], which enables LLMs to directly generate robot control code to achieve specific user goals. HumanEval benchmark [22] results indicate that LLMs demonstrate significant potential and flexibility in robotic task planning and control.

In the above studies, applying LLMs to robots is typically treated as a single intelligent agent. However, applying LLMs to dual-arm robots as a single intelligent agent often leads to issues such as decision-making errors and difficulties in executing parallel tasks. To address these challenges, this paper proposes a Dual-Arm Robot Multi-Agentization (DRMA) strategy based on LLMs. This approach divides a dual-arm nursing robot into multiple LLM-driven agents that collaborate to complete tasks. The inspiration for this strategy comes from multi-robot collaboration. Columbia University introduced ROCO [23], a multi-robot cooperation method that utilizes LLMs to facilitate communication, path planning, and collective reasoning among multiple robots, effectively enhancing coordination and planning. ROCO also demonstrates the advantages of the multi-robot cooperation method over centralized planning, including better adaptability, faster response times, and improved robustness in dynamic environments. MAD [24] adopts a similar approach by encouraging divergent thinking among multiple agents through debates. The framework effectively addresses the degeneration-of-thought problem by promoting “tit for tat” interactions between agents. Experimental results show that, compared to centralized planning in single-agent systems, multi-agent systems enable more powerful and creative reasoning. Ref. [25] uses multiple digital tools as multi-agents to assist in decision-making. Studies, such as Camel [26] and Chateval [27], have demonstrated that multi-agent systems based on LLMs significantly outperform single-agent systems in terms of performance. Multi-agent architectures provide distinct advantages in handling complex tasks and optimizing resource utilization, offering a novel pathway to overcoming the challenges faced by nursing robots.

The main contributions of this paper includes the following: (1) Proposing the LLM-based DRMA approach, which divides the left and right arms of a dual-arm robot into two intelligent agents that interact and collaborate through LLMs, thereby enhancing the overall task execution capability of the dual-arm nursing robot. (2) Developing a validation system that provides environmental feedback and error planning information, enabling the intelligent agents to optimize task planning and reduce time wasted on redundant planning. (3) Designing a trajectory planning algorithm based on an improved attractor model to enhance motion efficiency and adaptability. (4) Implementing and validating the DRMA approach by setting up two types of nursing task scenarios in the Mujoco simulation environment and one nursing task scenario in a real-world environment to demonstrate the effectiveness of both the DRMA strategy and the validation system.

## 2. DRMA

### 2.1. System Description

**Figure 1:**
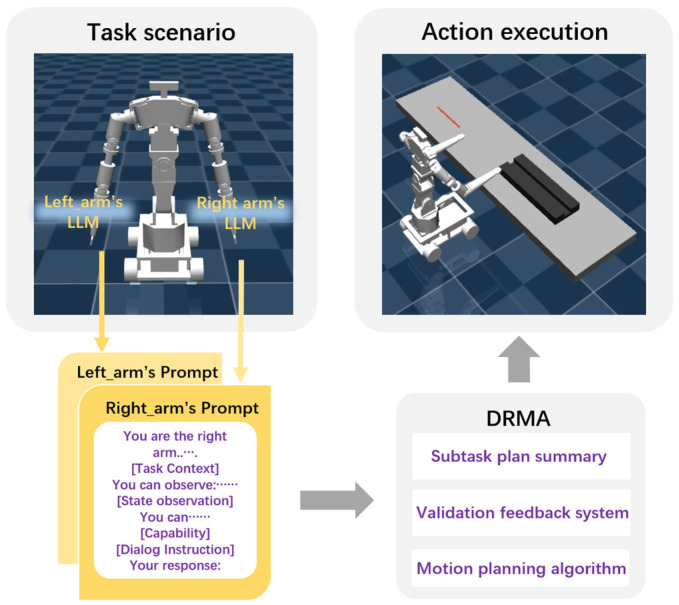
Overview of the dual-arm robot multi-agentization (DRMA) approach.

The left and right arms of the dual-arm nursing robot are positioned symmetrically on either side of its torso, as shown in Figure 1. Task planning must account for the coordinated movement of both arms with the torso and mobile base, with each arm selecting appropriate primitive skills based on the task requirements. During execution, the arms must avoid mutual interference and collisions, as well as obstacles in the environment.

In this study, the robot’s left arm and torso-base are designated as Agent 1, while the right arm and torso-base are designated as Agent 2, with each agent being assigned an independent LLM. This setup ensures that, while maintaining the robot as a single physical entity, it is treated as two independent agents at the communication and decision-making levels, each with distinct functions and task assignments. Although both agents include the torso-base component, a modular design for functions and tasks allows each agent to operate independently. Furthermore, the communication and information exchange between Agent 1 and Agent 2 enable them to share task progress and current states, allowing real-time adjustments to prevent conflicts and achieve task objectives.

**Figure 2:**
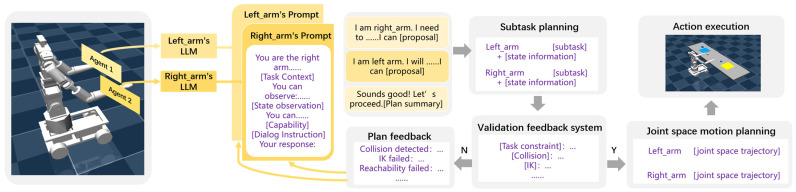
DRMA system.

During task execution, both arms must simultaneously account for the movement and posture of the torso-base, ensuring spatial coherence and coordination of the entire robot. This independent agent design facilitates distributed control, allowing each agent to focus on its designated tasks while collaborating to complete complex operations. The system structure is illustrated in Figure 2.

In a multi-agent system, each agent independently makes decisions and executes actions, enhancing system flexibility and adaptability. This design ensures that if one agent encounters a failure or requires adjustments, the rest of the system can continue functioning normally, maintaining task continuity and stability. The LLM-driven communication and coordination between agents enable them to assess each other’s needs and capabilities, ultimately improving the overall efficiency of the dual-arm robot through tight collaboration and optimized collective decision-making.

The DRMA framework consists of the following key components: LLM-driven agent dialogue mechanism, prompt engineering design, validation feedback system construction, and motion planning algorithm design.

### 2.2. LLM-Driven Agent Dialogue Mechanism

To address human–robot interaction challenges during task execution, this study designs an LLM-driven agent dialogue mechanism for dual-arm nursing robots. Each agent is equipped with an independent LLM, and before interacting with the environment, the agents engage in a preliminary dialogue through their respective LLMs. Each agent receives unique information specific to its role, such as the working skills and workspace of the left and right arms, and must respond according to predefined constraints (e.g., objects on the left side must be grasped by the left arm).

The LLM-based inter-agent dialogue must meet the following requirements: (1) One agent should be able to instruct another agent to continue the discussion or summarize the final steps required for execution, ultimately proposing a final action recommendation. (2) When summarizing the final action recommendations, the agents must adhere to the action format specified by prompt engineering and ensure that each agent has responded at least once within the dialogue.

These requirements ensure that agents can engage in structured discussions, allowing them to generate a task plan within a limited number of interactions in each round. The LLM-generated task plan is then mapped to predefined task-action parsing functions, ensuring that the robot accurately executes the intended actions.

### 2.3. Prompt Engineering Design

**Figure 3:**
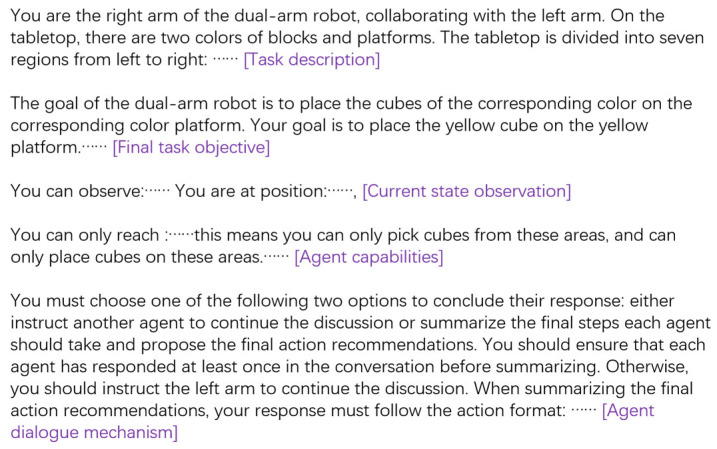
Prompt design for the right arm in the block sorting task.

Constructing appropriate language prompts can significantly enhance the language model’s understanding of task scenarios. To facilitate interaction between agents, each agent is assigned a unique name, allowing for smoother and more structured dialogues. Figure 3 illustrates the prompt design for the right arm in the block organization task, demonstrating how task instructions are formulated to optimize agent communication and execution.

When naming agents, a self-explanatory naming approach is adopted, where each agent is assigned a name that directly reflects its function (e.g., the agent controlling the left arm is named left_arm). Compared to anthropomorphic naming (e.g., naming the left-arm agent Jason) or sequential naming (e.g., naming it agent_1), this method enables the agent to better understand its own capabilities, state, and task context.

The language prompt design follows a shared overall structure, ensuring consistency across agents while allowing for task-specific adjustments based on each agent’s capabilities and state. The prompt includes the following key elements: (1) description of the current task scenario and final task objective, providing context for decision-making; (2) set of skills available to each agent within the given task, along with any operational constraints; (3) current state observations, offering real-time situational awareness; (4) guidelines on how agents should respond to dialogues initiated by other agents and how to format their responses correctly; and (5) reasons for the failure of the previous task plan (if applicable), allowing agents to refine their strategies accordingly.

This structured prompt design ensures that each agent accurately understands its role within the collaborative system, enhancing overall task efficiency and coordination.

### 2.4. Validation Feedback System Construction

To ensure the correct execution of tasks, it is essential to introduce a validation feedback mechanism within the multi-agent system. At the end of each dialogue round, the last agent to send a message is responsible for summarizing a task plan, which assigns a specific task to both arms for the current round. Before executing the assigned task, the validation feedback mechanism requires each agent to first verify the current task to ensure feasibility and correctness. The detailed validation process is outlined in Table 1, which specifies the key aspects each agent must assess before proceeding with execution.

The validation feedback process follows a structured sequence to ensure the correctness and feasibility of task execution. The verification steps proceed in the following order: (1) Task Format Validation: This step checks whether the generated task plan adheres to the required output format and includes all necessary keywords. Ensuring format correctness is crucial for subsequent validation steps. And this step also verifies whether the nature, objectives, and requirements of the assigned task conform to preset standards and align with the current round’s task demands. For example, if the task format does not include specific keywords such as “action execution”, the subsequent steps may not be able to correctly understand the task requirements or perform the corresponding actions. (2) Task–Capability Validation: This step ensures that the assigned task matches the capabilities of the respective agent. Different agents possess different skills and abilities, and if an agent is assigned a task beyond its operational limits, execution failure may occur. Additionally, this step checks whether the task complies with kinematic constraints, including joint limitations and the feasibility of the movement path. Through task capability validation, the system ensures that the task requirements align with the agent’s capabilities, thereby preventing execution failure. For example, the robotic arm’s workspace is limited to a certain area, and if the task requires the robotic arm to operate in an area it cannot reach, the task will fail. If the joint configuration returned by inverse kinematics for the target posture exceeds the robotic arm’s joint range, the target becomes unreachable, and the task plan must be readjusted. (3) Safety Validation: This step checks for potential collisions between robotic arms or between an arm and surrounding objects. By analyzing the target configuration, the system identifies possible collision risks, ensuring that the task can be executed safely without interference. For example, if the task paths of two robotic arms intersect during execution or a robotic arm interferes with an object in the environment, the safety validation will trigger a task plan readjustment to avoid collisions. These validation steps work sequentially, progressively refining the task plan to guarantee accuracy, feasibility, and safety in the execution of tasks by the multi-agent system.

The pseudocode for the system implementation process is presented in Algorithm 1, where $o_{t\;}$ represents the environmental observation in the current round; $\sigma_{t}$ denotes the robot’s target joint configuration; and $r_{t\;}$ represents the reward value, which evaluates whether the current task is successfully executed.
**Algorithm 1:** Multi-agent dialogue collaborationInput:agent 1 $a_{1}$, agent 2 $a_{2}\;$, Execution time range T; Input:Maximum replanning iterationsK; Feedback history Fh; t = 0 $o_{t}$ = env.initialize() **while** t < T **do**   Fh.clear_cache();   **while** len(Fh) < K **do**   plan = PlanSolution($a_{1},a_{2},Fh,o_{t}$);   valid_flag, feedback = validate_plan(plan)   **if** valid_flag:     executable_plan = plan;     **break**   **end if**    Fh.apped(feedback);   **end while**   **if** valid_flag:    $\sigma_{t}$ = MotionPlanning($o_{t}$, executable_plan)    $o_{t+1},r_{t+1}$ = env.step($\sigma_{t}$);    **if**  $r_{t+1}$ > 0:   **break**    **end if** **   end if**  t = t + 1 **end while**

If the current round’s task plan successfully passes the validation feedback, it will be combined with inverse kinematic (IK) solutions to generate the target configurations for both the left and right arms. These target configurations are then passed to the motion planner, which computes the corresponding motion trajectories for both arms to execute the task. If validation fails, the reason for failure will be recorded and added as feedback to the prompt for the next dialogue round, enabling the agents to re-plan based on the identified issues. When the re-planning attempts reach a predefined upper limit, the current round will terminate directly, and the system will proceed to the next round. If the task is not completed within the maximum allowed rounds, the entire task will be considered a failure.

### 2.5. Motion Planning Algorithm Design

The trajectory planning algorithm designed is based on an attractor model of dynamic movement primitives (DMPs) [28]. The algorithm conceptualizes the robot’s motion as the movement of a virtual mass under the influence of the attractor field in the DMP model. To optimize the trajectory and regulate joint velocity during motion, nonlinear function terms are introduced. Subsequently, a trajectory planning algorithm based on the improved attractor model is developed. This algorithm transforms the equivalent motion of the virtual mass in a virtual spring-damping system into a trajectory deviation, which is then mapped into the joint space using the Jacobian matrix transformation. The pseudocode is presented in Algorithm 2.
**Algorithm 2:** Trajectory Planning Algorithm Based on the Improved Attractor ModelInput:Initial joint angles $q_{0}$, Target pose g; Convergence threshold ε; Output:Planned trajectory $q_{plan}$ Obtain initial state:End-effector pose: y = Forwardkinematics($q_{0}$)          End-effector velocity: $\dot{y}=0$          Joint state: $q=q_{0}$, $\dot{q}=0$ **while True do:**   Pose deviation:d = ComputeError ($q,\dot{q},g$);   Acceleration under attractor model: $\ddot{y}=BaseAttraction(d,\dot{y})$   Constraint function term: F = LimitFunction ($d,\dot{y}$)   Attraction under improved attractor model: $f_{a}=ImprovedAttraction(\ddot{y},F)$   Pose acceleration mapped to joint space: $\ddot{q}=MapToJointspace(f_{a})$   $\dot{q}=\dot{q}+\ddot{q}dt$   $q=q+\dot{q}dt$   Update $q_{plan}$   $y,\dot{y}=Forwardkinematics(q,\dot{q})$  **if** d < ε:    **break**  **end if** **end while**

**Figure 4:**
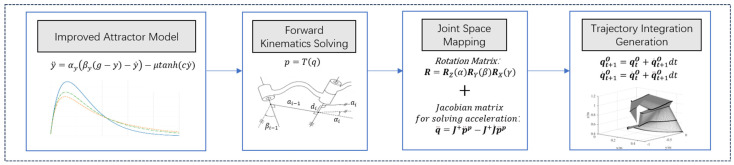
Flowchart of the overall algorithm implementation.

The implementation of Algorithm 2 relies on three key design parts: The attractor model based on dynamic movement primitives (DMP) provides a theoretical guarantee for trajectory convergence, ensuring stable motion planning. The attractor model is improved through the design of a nonlinear constraint function, optimizing trajectory performance and enforcing motion constraints. A trajectory planning algorithm based on the improved attractor model is developed, where the equivalent motion of a virtual mass under the influence of gravitational forces in a virtual spring-damping system is treated as trajectory deviation. This deviation is then transformed into joint space using the Jacobian matrix, generating the final planned trajectory. The flowchart of the overall algorithm implementation is shown in Figure 4.

The concept of the attractor model originates from DMP, which is an efficient method for generating robotic trajectories, first proposed by Schaal [20]. The core idea of this method is to construct an attractor model using a self-stabilizing second-order dynamic system, enabling the system to automatically converge to the attractor point under the driving force of dynamic equations, thereby achieving flexible control of trajectories. By changing the position of the attractor point, the final state of the system can be directly modified, allowing for adjustment of the trajectory’s target position. This method can be represented by the following second-order dynamic equation as its attractor model:

(1) $$\ddot{y}=\alpha_{y}\left(\beta_{y}\left(g-y\right)-\dot{y}\right)$$

In the above equation, $\alpha_{y}$ and $\beta_{y}$ are positive time constants; y represents the system state; $\dot{y}$ denotes the first-order derivative of y; $\ddot{y}$ denotes the second-order derivative of y; and the term g represents the target state.

Although the DMP attractor model inherently includes a damping mechanism through the linear damping term to suppress velocity growth and ensure convergence, its performance still shows certain limitations in practical tasks. In robotic arm trajectory planning, relying solely on linear damping may not fully meet the requirements for safety and trajectory smoothness. In particular, under certain operational scenarios, although linear damping can gradually attenuate velocity, there may still be instances where velocity peaks exceed safe limits within a short period. Such occurrences can not only lead to system oscillations but also pose operational risks for the robot. For example, when velocity becomes excessively high, linear damping may fail to effectively constrain joint motion, thereby increasing the risk of oscillation; during complex task transitions or when joint space constraints are present, depending only on linear attraction and damping forces may not suffice to ensure trajectory smoothness under highly dynamic environments.

Therefore, we propose an improved solution by introducing an additional constraint function $f\left(y,\dot{y}\right)$ into the attractor model, aimed at limiting the robot’s joint velocity and optimizing trajectory smoothness, formulated as follows:

(2) $$\ddot{y}=\alpha_{y}\left(\beta_{y}\left(g-y\right)-\dot{y}\right)+f(y,\dot{y})$$

The design of the function should satisfy the following conditions: (1) Continuity: The function must be continuous, and its first derivative must also be continuous. (2) Boundedness: The function must be bounded to guarantee system stability and prevent unbounded outputs. (3) Linear Damping Behaviors at Low Velocities: When the velocity is low, the function should exhibit linear damping characteristics, effectively acting as a standard damping term to regulate minor motions smoothly. (4) Rapid Saturation: The function should quickly approach saturation to limit further velocity growth, ensuring that the velocity remains within safe operational limits even under dynamic conditions.

Any function that satisfies the above conditions can be used as a candidate for designing this constraint function. Based on the analysis above, the hyperbolic tangent function tanh(x) and the arctangent function arctan(x) are selected as candidate constraint functions. The specific expression of the hyperbolic tangent function is calculated as follows:

(3) $$\begin{array}{c} \tanh\;(x)=\frac{e^{x}-e^{-x}}{e^{x}+e^{-x}} \end{array}$$

Both functions are continuous and bounded, exhibiting approximately linear behavior in the small input range while gradually saturating in the large input range, aligning with the previously established function design requirements. Compared to the arctangent function, the hyperbolic tangent function saturates more rapidly, allowing it to suppress velocity growth earlier, thereby enhancing system stability and safety. Additionally, the output range of the hyperbolic tangent function is (−1, 1), whereas the arctangent function has an output range of (−π/2, π/2). The normalized output range of the hyperbolic tangent function makes parameter tuning and processing more convenient, further improving its applicability in trajectory planning.

Based on the above analysis, this paper selects the hyperbolic tangent function (tanh(x)) as the constraint function. The specific constraint function $f\left(y,\dot{y}\right)$ is defined as follows:

(4) $$f\left(y,\dot{y}\right)=-\mu \tanh\;(c\dot{y})$$

In the above equation, μ and c are both constants, where μ controls the strength of the constraint function’s effect, and c determines the range of velocity over which the constraint function becomes active.

Depending on the magnitude of $\dot{y}$, the constraint function exhibits different effects and behaviors. When the velocity $\dot{y}$ is small ($\dot{y}\ll \frac{1}{c}$), the constraint function approximates a linear damping characteristic, expressed as follows:

(5) $$f\left(y,\dot{y}\right)\approx -\mu c\dot{y}$$

At this point, the constraint function effectively introduces an additional linear damping term. When the velocity $\dot{y}$ approaches the critical value ($\dot{y}\approx \frac{1}{c}$), the function exhibits nonlinear behavior and begins to exert a significant influence, effectively attenuating the output of the attractor model. This nonlinearity helps to limit excessive velocity growth, ensuring smoother and more stable trajectory execution.

When the velocity $\dot{y}$ becomes significantly large ($\dot{y}\gg \frac{1}{c}$), the constraint function approaches saturation, meaning the following:

(6) $$f\left(y,\dot{y}\right)\approx -\mu$$

At this stage, the constraint function forcibly attenuates acceleration, and its output tends toward a constant value. In general, this function primarily operates in the low-speed phase and the critical phase, ensuring smooth motion transitions and preventing abrupt velocity spikes. After introducing the constraint function $f\left(y,\dot{y}\right)$, the improved attractor model is formulated as follows:

(7) $$\ddot{y}=\alpha_{y}\left(\beta_{y}\left(g-y\right)-\dot{y}\right)-\mu \tanh\;(c\dot{y})$$

In the above equation, the constraint function not only limits velocity but also enhances trajectory smoothness and stabilizes velocity variations. By dynamically regulating acceleration and preventing abrupt changes in velocity, the function ensures that the robotic motion remains controlled, stable, and smooth throughout the trajectory execution.

**Figure 5:**
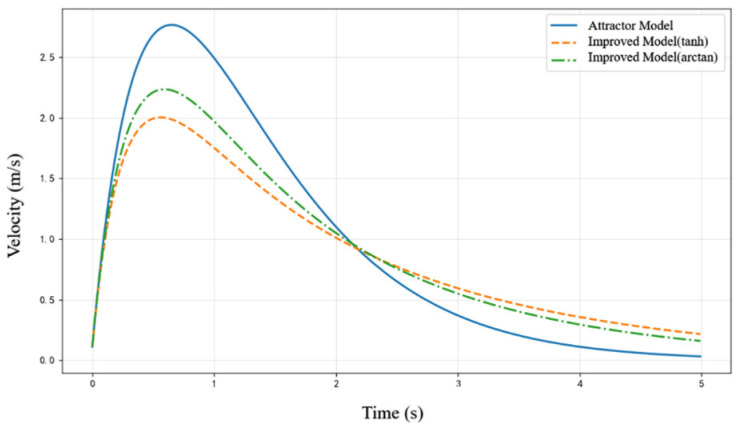
Comparison of velocity variation curves of different models during the convergence process.

To validate the effectiveness of the proposed improved attractor model in velocity limitation and trajectory smoothness, a verification scenario was constructed. In this simulation, the system’s motion from the initial position y=0 to the target position g=5 was analyzed, comparing the original attractor model with the improved attractor model. The parameters were set as follows: $\alpha_{y}=3$, $\beta_{y}=0.75$. To ensure that the maximum velocity during convergence does not exceed 2.5 m/s, the constraint function parameters were set as: μ=5, c=0.4. The velocity variation during the convergence process of the improved attractor model is illustrated in Figure 5. Additionally, the figure also presents the velocity variation when using the standardized arctan function as the constraint function.

From Figure 5, it can be observed that compared to the original attractor model, the improved attractor model with the constraint function effectively limits the maximum velocity during the convergence process. Additionally, it ensures smoother velocity transitions, preventing instability or potential energy losses caused by excessive velocity. Using the improved model, although both constraint functions effectively regulate velocity, the nonlinear characteristics of the tanh function provide a more pronounced limitation in high-velocity regions. This results in smaller fluctuations in the velocity curve and smoother velocity transitions, demonstrating superior stability. The experimental results confirm that by introducing the constraint function, the improved model not only reduces the peak velocity during convergence but also enhances overall velocity smoothness, leading to more stable and controlled trajectory execution.

To ensure the safe execution of tasks by the dual-arm robot, this study proposes a trajectory planning algorithm based on the improved attractor model. First, the improved attractor model is used to calculate the attractive force exerted by the target pose on the robot in Cartesian space. Then, a virtual spring-damping model is introduced, treating the equivalent motion of the virtual spring-damping system under the influence of this attractive force as the trajectory deviation. Finally, the trajectory deviation is transformed into joint space using the Jacobian matrix.

Assuming that ${\left\{q_{t}^{d},{\dot{q}}_{t}^{d},{\ddot{q}}_{t}^{d}\right\}}_{t=0}^{T}$ represents the planned joint-space trajectory for the redundant robotic arm, the corresponding planned pose trajectory ${\left\{p_{t}^{d},{\dot{p}}_{t}^{d}\right\}}_{t=0}^{T}$ can be obtained through forward kinematics and Jacobian matrix transformation. To ensure that the trajectory motion both meets the planned target and adapts to the environment in real time, the end-effector reference velocity ${\dot{p}}_{t}^{c}$ of the robotic arm can be expressed as follows:

(8) $${\dot{p}}_{t}^{c}={\dot{p}}_{t}^{d}+{\dot{p}}_{t}^{I}$$

In the above equation, ${\dot{p}}_{t}^{d}$ represents the planned velocity, while ${\dot{p}}_{t}^{I}$ denotes the dynamically adjusted velocity, which is used to correct the planned velocity to ensure real-time adaptability to environmental changes.

To achieve accurate tracking of the end-effector pose trajectory, it is necessary to compute the deviation between the reference pose and the current pose. Due to the redundancy of the robotic arm, multiple joint configurations can result in the same end-effector pose, leading to the issue where the orientation representation of specific poses is not unique. This non-uniqueness affects the computation of the corrected reference pose $p_{t}^{c}$, making it impossible to obtain directly through simple summation. To address this issue, this study introduces rotation matrices to represent pose deviations. By using the difference between rotation matrices, the uniqueness and non-singularity of rotation matrices help to avoid ambiguities in certain orientation representations, ensuring a more robust and consistent correction of the reference pose.

In this study, the rotation matrix based on Euler angles is used to represent the robot’s orientation. The Euler angles are defined by sequential rotations around the *Z*-axis, *Y*-axis, and *X*-axis, denoted as α, β and γ, respectively. The corresponding rotation matrix is given by the following formula:

(9) $$R=R_{Z}\left(\alpha \right)R_{Y}\left(\beta \right)R_{X}\left(\gamma \right)\;=\left[\begin{array}{ccc} c\alpha & -s\alpha & 0\\ s\alpha & c\alpha & 0\\ 0 & 0 & 1 \end{array}\right]\left[\begin{array}{ccc} c\beta & 0 & s\beta \\ 0 & 1 & 0\\ -s\beta & 0 & c\beta \end{array}\right]\left[\begin{array}{ccc} 1 & 0 & 0\\ 0 & c\gamma & -s\gamma \\ 0 & s\gamma & c\gamma \end{array}\right]\;=\left[\begin{array}{ccc} c\alpha c\beta & c\alpha s\beta s\gamma -s\alpha c\gamma & c\alpha s\beta c\gamma +s\alpha s\gamma \\ s\alpha c\beta & s\alpha s\beta s\gamma +c\alpha c\gamma & s\alpha s\beta c\gamma -c\alpha s\gamma \\ -s\beta & c\beta s\gamma & c\beta c\gamma \end{array}\right]$$

The homogeneous transformation matrix representing the reference pose $p^{c}$ relative to the base coordinate system is given by the following:

(10) $$T_{c}^{O}=T\left(p^{d}\right)T\left(p^{I}\right)=\left[\begin{array}{cc} R_{d}^{O}R_{I}^{O} & {\left(X_{d}^{O}+X_{I}^{O}\right)}^{T}\\ 0^{1\times 3} & 1 \end{array}\right]$$

The homogeneous transformation matrix representing the reference pose $p^{c}$ relative to the current pose $p^{p}$ is given by the following:

(11) $$T_{c}^{p}={\left(T_{p}^{O}\right)}^{-1}T_{c}^{O}$$

And the rotation matrix corresponding to the homogeneous transformation matrix $T_{c}^{p}$ is given by the following:

(12) $$R_{c}^{p}=T\left[1:3,1:3\right]$$

The attitude angles $W_{c}^{p}$ can be extracted from the rotation matrix $R_{c}^{p}$.

(13) $$W_{c}^{p}=\left[\begin{array}{c} arctan2\;(R_{c,21}^{p},R_{c,11}^{p})\\ arcsin\;({-R}_{c,31}^{p})\\ arctan2\;(R_{c,32}^{p},R_{c,33}^{p}) \end{array}\right]$$

Then, at any given moment, the deviation between the reference pose and the current pose of the robotic arm’s end-effector in the current coordinate frame $R_{p}^{O}$ is as follows:

(14) $$d_{p}\left(p^{c},p^{p}\right)={vec\left\{\left[X_{c}^{p},W_{c}^{p}\right]\right\}}^{T}$$

The above equation represents the process where the current end-effector pose of the robotic arm first translates by $X_{c}^{p}$ along the current coordinate frame $R_{p}^{O}$, and then rotates by $W_{c}^{p}$ around $R_{p}^{O}$. Meanwhile, to convert pose velocity into joint velocity, it is necessary to compute the mapping of $d_{p}\left(p^{c},p^{p}\right)$ in the base coordinate system.

(15) $$d_{O}\left(p^{c},p^{p}\right){=vec\left\{\left[{R_{O}^{p}X}_{c}^{p},{R_{O}^{p}W}_{c}^{p}\right]\right\}}^{T}$$

The motion of the robot can be regarded as the movement of a virtual mass under the attraction force of the improved attractor model. The attraction force $f_{a}$ of the improved attractor model can be obtained from the following equation:

(16) $$f_{a}=\sigma \left[\alpha_{y}\left(\beta_{y}\left(g-y\right)-\dot{y}\right)-\mu tanh\;(c\dot{y})\right]$$

In the above equation, σ is a scaling coefficient used to adjust the mapping relationship. The attraction force $f_{a}$ not only includes the linear attraction force and damping force from the traditional attractor model but also incorporates a nonlinear correction term, which enhances the control over both the trajectory and velocity of the virtual mass. This attraction force ensures a smooth trajectory deviation with continuous acceleration. Assuming the positive definite inertia matrix of the virtual mass is $M_{O}\in R^{6\times 6}$, the acceleration of the end-effector is given by the following:

(17) $${\ddot{p}}^{p}={\left(M_{O}\right)}^{-1}[\begin{array}{c} f_{a}\\ 0^{3*1} \end{array}]$$

Using the Moore–Penrose pseudoinverse method to compute the inverse of the Jacobian matrix, the pose acceleration can be transformed into the joint space as follows:

(18) $$\ddot{q}=J^{+}{\ddot{p}}^{p}-J^{+}\dot{J}{\ddot{p}}^{p}$$

By discretizing the above equation, the joint angle trajectory deviation at any given time can be expressed as follows:

(19) $$\left\{\begin{array}{l} q_{t+1}^{O}=q_{t}^{O}+{\dot{q}}_{t+1}^{O}dt\\ {\dot{q}}_{t+1}^{O}={\dot{q}}_{t}^{O}+{\ddot{q}}_{t+1}^{O}dt\\ {\ddot{q}}_{t+1}^{O}=\ddot{q} \end{array}\right.$$

## 3. Experiments and Results

### 3.1. Experimental Setup

Each agent is equipped with the GLM-4 [29] model. In addition to the multi-agent dialogue collaboration method, a single-agent planning mode is also set up for comparison. In the single-agent planning mode, a central planner equipped with an LLM receives environmental information as well as the capability data of both arms. It then uses the same validation feedback system to plan tasks for the left and right arms.

Additionally, control groups without feedback are set up for both the multi-agent and single-agent modes (the multi-agent mode is referred to as the DRMA mode). The hardware configuration used in this experiment is shown in Table 2.

This study designs two experimental tasks: placing the rope and organizing blocks. Successfully completing these tasks requires three types of collaboration modes: simultaneous grasping or placing by both arms, object exchange between the left and right arms, and independent operation of each arm.

### 3.2. Task Scenarios

**Figure 6:**
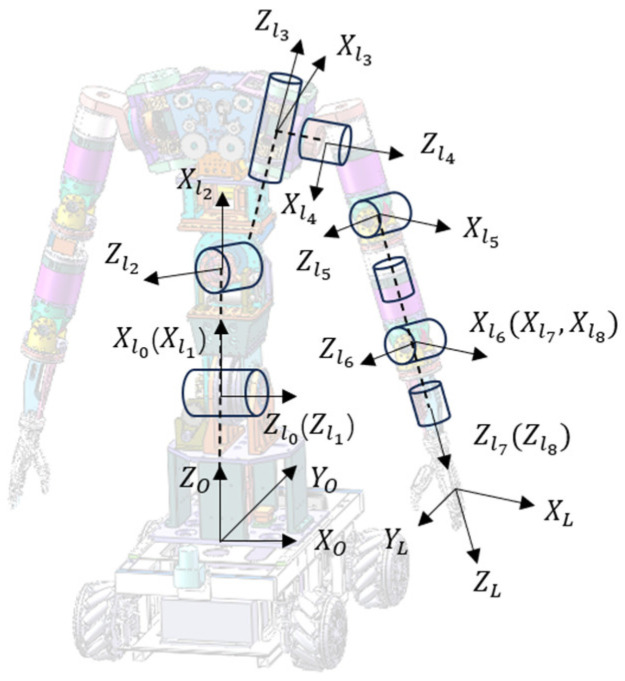
Kinematic modeling of the dual-arm robot.

In this study, SolidWorks 2020 and the Mujoco 2.3.7 physics engine were used to construct the experimental models and scenarios. In constructing the kinematic model, the dual-arm robot is equivalently treated as two redundant eight-degree-of-freedom serial mechanisms, and the kinematic model of the dual-arm robot is expressed using the improved D-H parameter method. Since the two robotic arms have identical structures and exhibit symmetry in spatial layout, for the sake of simplifying the analysis process, the kinematic model of the left arm of the dual-arm robot is used as an example. Based on the improved D-H parameter principles, the established robot coordinate system is shown in Figure 6.

The origin of the robot’s base coordinate system is defined at the intersection of the perpendicular line between the mobile chassis and the torso joints. The modified D-H parameters for the dual-arm robot are shown in Table 3. In the table, $\alpha_{i}$ represents the joint angle under different coordinate systems, $d_{i}$ represents the link offset distance under different coordinate systems, $a_{i-1}$ represents the link length under different coordinate systems, and $\beta_{i-1}$ represents the link twist angle under different coordinate systems. To complete the control strategy for the dual-arm robot, a model-based control method is employed to ensure the precise coordination between the two arms during motion. Based on the aforementioned kinematic model, the structure and dynamic parameters of the robotic arm can be described using an Extensible Markup Language (XML) file, which allows for simulation and control using the Mujoco physics engine.

**Figure 7:**
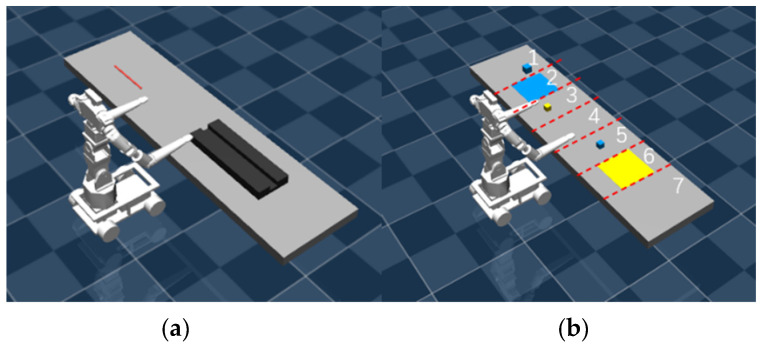
Task scenario diagram. (**a**) Placing the rope task scenario. (**b**) Organizing blocks task scenario.

The scenarios include placing the rope and organizing blocks in the task environments, as shown in Figure 7. Figure 7a illustrates the placing a rope task scenario, where the robot must manipulate and position a flexible rope accurately. Figure 7b depicts the organizing blocks task scenario, requiring the robot to sort and arrange scattered blocks. For object manipulation, the robot uses a suction-based gripping mechanism to pick up and place items efficiently.

Task 1: Placing the Rope. In this task, the robot’s left and right arms must grasp both ends of the rope and accurately place it into a designated groove while avoiding collisions between the two arms during grasping and placement.

Capabilities and Goals: Both arms can pick up and place either end of the rope within their reachable range.

Collaboration Mode: The left and right arms must simultaneously grasp and place the rope.

Task 2: Organizing Blocks. In this scenario, the workspace is divided into seven regions from left to right, containing two types of blocks and two platforms, as shown in Figure 7b. The robot’s objective is to sort and place each block type onto the corresponding platform. If a block is outside the reach of a robotic arm, the other arm must assist by transferring it through a shared central area.

Capabilities and Goals: The left arm places Type 1 blocks (Block 1, Block 2) onto Platform 1. Its operational range is limited to the left-side area of Platform 1, the platform itself, its right-side area, and the upper part of the central shared area. The right arm places Type 2 blocks (Block 3) onto Platform 2. Its operational range is limited to the left-side area of Platform 2, the platform itself, its right-side area, and the upper part of the central shared area. The two arms must cooperate to transfer blocks through the central shared area if a block is outside their individual reach.

Collaboration Mode: object exchange between the left and right arms; independent operation of each arm.

### 3.3. Task Planning Simulation Experiments

**Figure 8:**
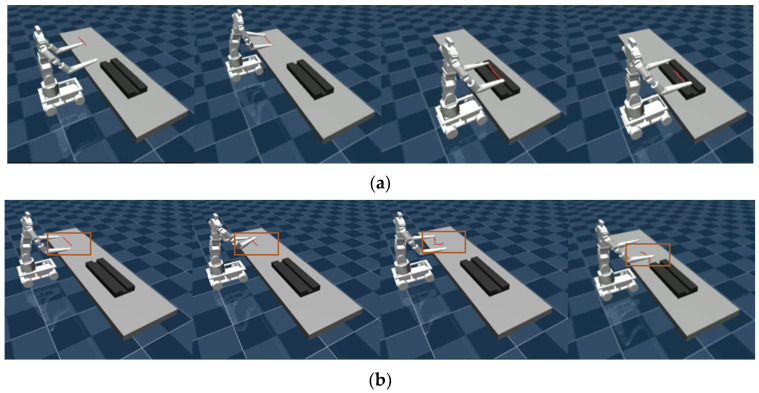
Schematic diagram of task operation. (**a**) Successful steps in placing the rope. (**b**) Failure scenario in placing the rope.

In the placing the rope task, the robot’s left and right arms simultaneously grasp and place the rope, as shown in Figure 8a. The failure cases include the robot failing to locate both ends of the rope, both arms attempting to grasp the same end simultaneously, only one arm successfully grasping the rope, or failing to place it in the designated position, as illustrated in Figure 8b.

**Figure 9:**
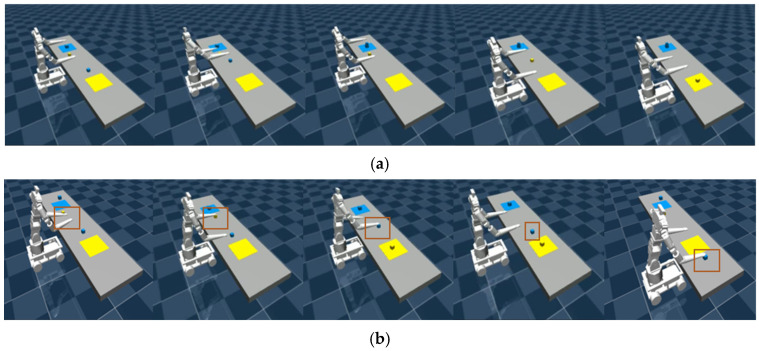
Schematic diagram of task operation. (**a**) Successful steps in organizing the blocks. (**b**) Failure scenario in organizing the blocks.

In the organizing blocks task, the optimal planning steps are as follows: First, the left arm picks up Block 1 from the left-side area of Platform 1 and places it in the Platform 1 area. Second, the right arm picks up Block 2 from the left-side area of Platform 2 and places it in the central shared area. Third, the left arm picks up Block 2 from the central shared area and places it in the Platform 1 area. Fourth, the left arm picks up Block 3 from the right-side area of Platform 1 and places it in the central shared area. Fifth, the right arm picks up Block 3 from the central shared area and places it in the Platform 2 area, as shown in Figure 9a. By coordinating the dual-arm actions, unnecessary grasping and placement operations are minimized while fully utilizing the central shared area as a transfer point, achieving optimized task efficiency. Figure 9b illustrates a failure case in the task.

For both the placing a rope and organizing blocks tasks, each task was executed for 20 rounds in each experimental group. The average task success rate (ASR), average number of steps (ANS), and average number of planning iterations (ANP) were recorded as the three evaluation metrics, as shown in Table 4.

The results indicate that in the feedback-enabled condition, the multi-agent mode outperforms the no-feedback mode in all task evaluation metrics. In the single-agent mode, when no feedback is available, the agent may persist in faulty plans (e.g., both arms attempting to grasp the leftmost and rightmost blocks simultaneously), leading to task failures and reduced efficiency. However, when feedback is introduced, the agent can adjust and optimize its plan based on the feedback. Although this increases the number of planning iterations, it significantly improves the task success rate (e.g., in the organizing blocks task, the success rate increased from 20% to 70%). In the multi-agent mode, agents can collaborate and share information, optimizing task execution by reducing unnecessary re-planning and error corrections. As a result, the feedback system actually reduces the average number of planning iterations in this mode. Additionally, under the 10-step limit imposed on the average number of task steps, the no-feedback mode performs only one planning step per iteration. This causes the task to terminate once the step limit is reached, effectively stopping further planning. If the step limit were increased, the number of planning iterations in the no-feedback mode would continue to rise, further widening the gap between feedback and no-feedback conditions. The feedback system helps agents optimize their plans through environmental feedback, improving success rates and efficiency while reducing errors and minimizing wasted time.

Comparing the performance of single-agent and multi-agent collaboration across tasks, both modes achieve similar success rates (only a 10% higher success rate for multi-agent in the placing rope task). However, multi-agent collaboration shows significant advantages in step efficiency and planning efficiency. In single-agent mode, due to global optimal planning, the system may overlook physical constraints (e.g., the left and right arms cannot simultaneously grasp the leftmost and rightmost blocks). In tasks like organizing blocks, which involve multiple steps and multiple objectives, such erroneous planning occurs repeatedly. In contrast, in multi-agent mode, agents can use dialogue-based interaction to develop cooperative strategies and exchange information in real time during execution to adjust their strategies dynamically. This collaboration mechanism effectively reduces unnecessary steps and redundant planning iterations caused by incorrect plans in the single-agent mode, thereby enhancing adaptability to the environment. This cooperative approach effectively avoids the planning pitfalls of single-agent mode, making it particularly effective in complex tasks such as multi-objective block sorting.

### 3.4. Trajectory Generation Simulation Experiments

To verify the effectiveness of the trajectory planning algorithm based on the improved attractor model proposed in this study, experiments were conducted in the Mujoco simulation environment, combined with MATLAB R2023b’s Robotics Toolbox for data analysis and trajectory planning validation. The proposed algorithm was applied to the model, and the simulated robotic arm’s movements were generated. In order to further enhance the smoothness of the generated trajectory, polynomial interpolation techniques were employed, which can provide smooth transitions between key points [30,31]. Additionally, a comparison of joint velocity variations was conducted under the RRT model, the standard attractor model, and the improved attractor model.

The virtual positive definite inertia matrix is set as: $M_{O}=diag(\left[1,1,1,1,1,1\right]\times 10^{3})$, with the following parameters: $\sigma =10^{-3}$, $\alpha_{y}=4$, $\beta_{y}=0.25$, μ=0.5, c=1. The initial posture of the dual-arm robot is as follows:

(20) $$\left\{\begin{array}{c} q_{l}={\left[0,\frac{7\pi }{12},\frac{\pi }{2},-\frac{\pi }{2},\frac{2\pi }{3},0,0,0\right]}^{T}\\ q_{r}={\left[0,\frac{5\pi }{12},-\frac{\pi }{2},-\frac{\pi }{2},\frac{2\pi }{3},0,0,0\right]}^{T} \end{array}\right.$$

where, $q_{l}$ represents the initial posture of the robot’s left arm, while $q_{r}$ represents the initial posture of the robot’s right arm.

**Figure 10:**
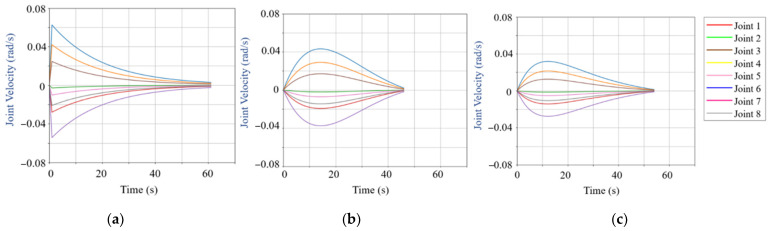
Joint velocity variation curves under different models. (**a**) Joint velocity variation curves of RRT model. (**b**) Joint velocity variation curves of attractor model. (**c**) Joint velocity variation curves of improved attractor model.

Based on the above parameter settings, this study compares the joint velocity variations when using the RRT model optimized with a virtual force field, the attractor model, and the improved attractor model. The joint velocity variation graphs under different models are shown in Figure 10.

Figure 10 illustrates the joint velocity variations of the right-arm redundant manipulator during motion execution under different models. Comparing Figure 9a and Figure 10b, the attractor model effectively mitigates the sharp velocity spikes and abrupt changes present in the original RRT model, resulting in smoother velocity transitions. Further comparing Figure 10b,c, it is evident that the improved attractor model, incorporating the constraint function, further limits the peak velocity of the redundant manipulator during execution. The greater the peak velocity in the motion process, the more significant the velocity-limiting effect of the improved attractor model. Additionally, the velocity transitions in the improved attractor model are smoother compared to the standard attractor model, demonstrating enhanced motion stability.

**Figure 11:**
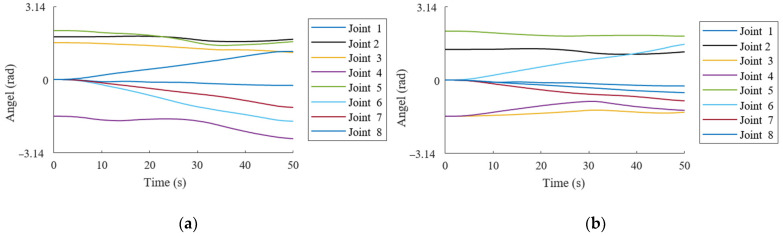
Joint position variation during the robotic arm grasping process. (**a**) Left arm joint position variation during grasping. (**b**) Right arm joint position variation during grasping.

To analyze the motion characteristics of the trajectory planning algorithm based on the improved attractor model during the robotic arm’s grasping process, it is necessary to record the joint position variations. This evaluation helps assess the specific optimization effects of the improved attractor model on trajectory planning. As shown in Figure 11, the joint position variations during the grasping process exhibit smooth transitions without abrupt jumps or drastic fluctuations, demonstrating good trajectory continuity.

**Figure 12:**
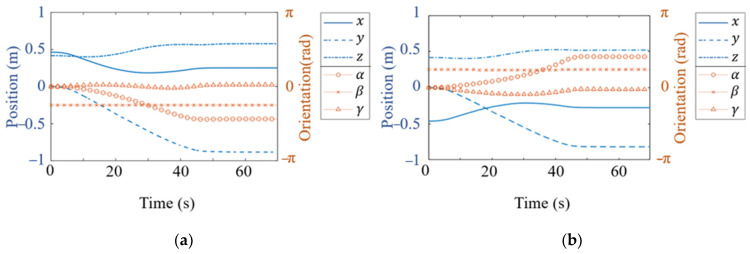
Robotic arm grasping pose trajectory. (**a**) Left arm grasping pose trajectory. (**b**) Right arm grasping pose trajectory.

Using Equation (13), the end-effector pose of the dual-arm robot can be computed. The planned end-effector pose trajectory is shown in Figure 12.

From Figure 12, it can be observed that the planned joint angles and end-effector pose trajectories are continuous and smooth. The trajectory planning algorithm based on the improved attractor model generates a smooth and continuous pose trajectory, effectively avoiding sudden jerks and discontinuous changes. This ensures that the robot exhibits more stable motion characteristics during task execution, improving both trajectory controllability and execution accuracy.

**Figure 13:**
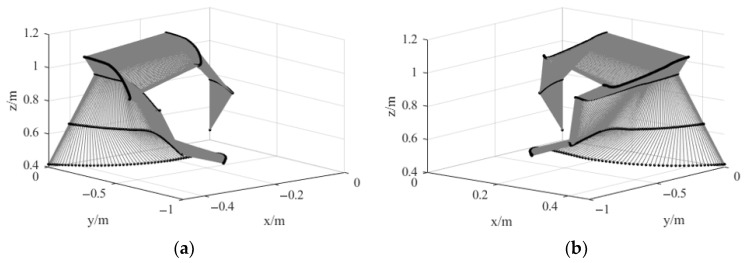
Left and right arm grasping planning trajectory of the robotic arm. (**a**) Left arm grasping planned trajectory. (**b**) Right arm grasping planned trajectory.

The joint trajectory curves corresponding to the end-effector’s motion along the planned pose trajectory in the workspace are shown in Figure 13. Figure 13 illustrates how the robotic arm joints iteratively adjust their trajectories during the grasping motion using the attractor model. The end-effector pose successfully converges to the desired pose, and the resulting trajectory remains continuous and smooth, without abrupt changes.

Therefore, the simulation experiments validate the effectiveness of the trajectory planning algorithm based on the improved attractor model. The results confirm that the algorithm can generate smooth and oscillation-free trajectories, effectively addressing issues of sudden joint velocity changes and velocity spikes in trajectory planning tasks. Additionally, the algorithm demonstrates its capability to accurately execute the action sequences planned by the large language model.

### 3.5. Real-World Experiments

The simulation experiments validated the performance of the DRMA method in planning and executing the block-organizing task, particularly in scenarios where the two blocks were not simultaneously within the dual-arm’s reachable range.

**Figure 14:**
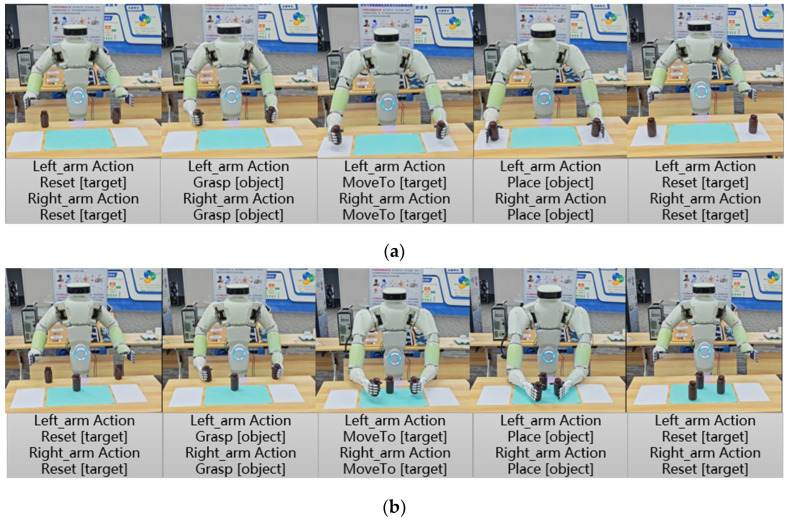
Real-world experiment of the medicine bottle sorting. (**a**) Real-world experiment for bottle sorting task 1. (**b**) Real-world experiment for bottle sorting task 2.

To further verify the effectiveness of the DRMA method, this section conducts real-world experiments using a bottle-organizing scenario. The experiment is performed on a physical robot, covering a sequence of operations from grasping to moving, placing, and resetting, as illustrated in Figure 14. The dual-arm robot is capable of executing the following actions: “Grasp”, ”Move To”, ”Place”, ”Reset”, and “Wait”.

The experiment tasks are divided into two types: one is classifying and placing two medicine bottles into separate designated areas on both sides, and another is placing both medicine bottles in a central area. The execution process follows these steps: The dual arms first reset to the initial position, then grasp the medicine bottles, move them to the designated location, and place them down. Finally, the arms return to the initial position, completing the task.

To validate the effectiveness of the DRMA method, 10 trials were conducted to compare the single-agent method and the DRMA method based on average number of task success rate, average number of steps, average number of planning iterations, and average number of execution time (AET). The results are presented in Table 5.

The DRMA method achieved a 100% success rate in both tasks, while the single-agent method reached only 90% success in the second task, demonstrating that DRMA ensures greater task execution stability.

In terms of efficiency, the DRMA method significantly reduced the number of execution steps and total execution time. Compared to the single-agent method, the average number of steps was reduced by approximately 29%, and the average execution time was shortened by about 28%, highlighting its higher task execution efficiency.

The low efficiency of the single-agent method can be attributed to its failure to adequately consider the limitations of the dual-arm operating space and grasping range, as well as the constraints in planning decisions. For example, in one failure case, the single-agent method planned for both arms to simultaneously grasp medicine bottles outside their respective operating areas, ignoring the grasping range limitations, which led to planning failure and increased execution time. In another failure case, the single-agent method planned for the left arm to grasp and the right arm to place, but the right arm had not yet grasped the bottle, resulting in failure. This highlights the lack of synchronization between the left and right arms in the single-agent method.

During re-planning, the single-agent method failed to plan for the left and right arms to grasp different bottles separately, instead opting for a sequence where the left arm grasped and the right arm waited. Although this allowed the task to progress, it increased the number of steps and execution time. The single-agent method tends to select safer but suboptimal solutions during re-planning, relying more on single-arm operation and failing to make full use of dual-arm collaboration, leading to inefficient planning and failure to generate the optimal solution.

These issues are particularly evident in multi-step tasks, contributing to unstable planning quality and increased task failure risks. The DRMA method, by equipping each arm with an independent large language model agent and incorporating an intelligent dialogue mechanism and prompt engineering, addresses the issues of inefficient task allocation and redundant steps seen in the single-agent method. This enables the dual-arm robot to generate an optimal five-step operation plan.

## 4. Conclusions

This paper proposes an LLM-based dual-arm nursing robot multi-agentization (DRMA) method, which treats the left and right arms of the dual-arm nursing robot as independent agents, each equipped with an LLM to enable interaction and coordination between agents, thereby improving the robot’s task execution efficiency and success rate. A validation feedback mechanism is established, providing environmental feedback and error planning information to allow the agents to optimize task planning. A trajectory planning algorithm based on the improved attractor model is designed, enabling the robot to effectively execute action sequences generated by the validation feedback system. Simulation experiments were conducted in the Mujoco environment, followed by real-world experiments to validate the method. The results show that the DRMA method, through agent interaction and cooperation along with the introduction of the validation feedback mechanism, not only increases the success rate of task execution but also reduces the number of planning steps, improving task completion efficiency. Future research will prioritize learning-based prompts to enhance agent adaptability and decision-making flexibility in dynamic environments, alongside integrating real-time vision systems (e.g., RGB-D cameras) to improve robotic perception and safe interaction capabilities in nursing scenarios.

## Figures and Tables

**Table 1:** Verification content of the feedback verification system.

Verification Content	Description
Task Format Validation	Ensures task plan format compliance and execution-round alignment.
Task–Capability Validation	Validates task alignment with agent capabilities.
Safety Validation	Checks posture achievability via inverse kinematics and collision risks during execution.

**Table 2:** Experimental environment setup.

Component	Configuration Information
Operating System	Windows 11
CPU	AMD Ryzen 9 5900HX
GPU	NVIDIA GeForce RTX 3090
RAM	32 GB
Programming Language	Python 3.8.18

**Table 3:** D-H parameters of the dual-arm robot.

$Link{\;l}_{i}$	$\alpha_{i}$ /rad	$d_{i}$ /cm	$a_{i-1}$ /cm	$\beta_{i-1}$ /rad
$l_{1}$	$\alpha_{1}$	0	0	0
$l_{2}$	$\alpha_{2}$	0	$a_{2}$	−π/2
$l_{3}$	$\alpha_{3}$	$d_{3}$	$a_{3}$	π/2
$l_{4}$	$\alpha_{4}$	$d_{4}$	0	π/2
$l_{5}$	$\alpha_{5}$	0	$a_{5}$	π/2
$l_{6}$	$\alpha_{6}$	$d_{6}$	0	π/2
$l_{7}$	$\alpha_{7}$	0	0	−π/2
$l_{8}$	$\alpha_{8}$	$d_{8}$	0	π/2

**Table 4:** Summary of evaluation metrics results for simulation experiment tasks.

Task Type	Agent Mode	Feedback Used	ASR	ANS	ANP
Placing the Rope	Single-Agent	Yes	90%	3.2	5.2
Placing the Rope	Single-Agent	No	70%	4.6	4.6
Placing the Rope	DRMA	Yes	100%	2.4	2.9
Placing the Rope	DRMA	No	80%	3.8	3.8
Organizing Blocks	Single-Agent	Yes	70%	7.1	12.0
Organizing Blocks	Single-Agent	No	20%	9.3	9.3
Organizing Blocks	DRMA	Yes	70%	6.5	6.8
Organizing Blocks	DRMA	No	70%	6.9	6.9

**Table 5:** Summary of evaluation metrics for real-world experiment tasks.

Task Type	Agent Mode	ASR	ANS	ANP	AET
Bottle Sorting Task 1	Single-Agent	100%	6.8	7.8	216.86 s
Bottle Sorting Task 1	DRMA	100%	5.0	5.7	157.46 s
Bottle Sorting Task 2	Single-Agent	90%	7.3	8.9	228.61 s
Bottle Sorting Task 2	DRMA	100%	5.0	5.8	162.32 s

## Data Availability

Data are contained within the article.
